# Supplementary material for: Perinatal mortality in German dairy cattle: Unveiling the importance of cow-level risk factors and their interactions using a multifaceted modelling approach
Source: PLoS One. 2024 Apr 17;19(4):e0302004. doi: 10.1371/journal.pone.0302004 (PMC11023303; doi:10.1371/journal.pone.0302004)
Supplement: S1 File — (DOCX) [file pone.0302004.s001.docx]

Supplementary material

Table of Contents

# 1 Model without interactions

## 1.1 R code

m_multi_no_int <- glmer(
 PM ~ breed + calving + farm_size + parity + season + (1 | farm_id),
 data = d, family = binomial(),
 control = glmerControl(optimizer ='bobyqa', optCtrl=list(maxfun=1e6)))

## 1.2 Equation

extract_eq(m_multi_no_int, wrap = TRUE, ital_vars = TRUE)

$$\begin{matrix} PM_{i} & \sim\mathrm{Binomial}\left( n=1,\mathrm{prob}_{PM=1}=\hat{P} \right) \\ \log\left[ \frac{\hat{P}}{1-\hat{P}} \right] & =\alpha_{j\left[ i \right]}+\beta_{1}\left( breed_{SIM} \right)+\beta_{2}\left( breed_{BS} \right)+\beta_{3}\left( breed_{others} \right) + \\ & \beta_{4}\left( calving_{medium} \right)+\beta_{5}\left( calving_{difficult} \right)+\beta_{6}\left( parity_{2} \right)+\beta_{7}\left( parity_{3+} \right) + \\ & \beta_{8}\left( season_{autumn} \right)+\beta_{9}\left( season_{winter} \right)+\beta_{10}\left( season_{spring} \right) \\ \alpha_{j} & \sim N\left( \gamma_{0}^{\alpha}+\gamma_{1}^{\alpha}\left( farm\_size_{medium} \right)+\gamma_{2}^{\alpha}\left( farm\_size_{large} \right),\sigma_{\alpha_{j}}^{2} \right)\text{, for farm\_id j = 1,}\ldots\text{,J} \end{matrix}$$

Where:

- p - represents the probability of the outcome
- α – intercept
- β – regression coefficient
- (1|farm_id) – the random effect on the intercept
- ϵ - error term

# 2 Model with four important interactions

## 2.1 R code

model <- glmer(
 PM ~ breed*calving + parity*season + breed*season + farm_size*calving + (1 | farm_id),
nAGQ = 0, data = d, family = binomial(),
control = glmerControl(optimizer ='bobyqa', optCtrl=list(maxfun=1e6)))

## 2.2 Equation

extract_eq(model, wrap = TRUE, ital_vars = TRUE)

$$\begin{matrix} PM_{i} & \sim\mathrm{Binomial}\left( n=1,\mathrm{prob}_{PM=1}=\hat{P} \right) \\ \log\left[ \frac{\hat{P}}{1-\hat{P}} \right] & =\alpha_{j\left[ i \right]}+\beta_{1}\left( breed_{SIM} \right)+\beta_{2}\left( breed_{BS} \right)+\beta_{3}\left( breed_{others} \right) + \\ & \beta_{4}\left( calving_{medium} \right)+\beta_{5}\left( calving_{difficult} \right)+\beta_{6}\left( parity_{2} \right)+\beta_{7}\left( parity_{3+} \right) + \\ & \beta_{8}\left( season_{autumn} \right)+\beta_{9}\left( season_{winter} \right)+\beta_{10}\left( season_{spring} \right)+\beta_{11}\left( breed_{SIM}\times calving_{medium} \right) + \\ & \beta_{12}\left( breed_{BS}\times calving_{medium} \right)+\beta_{13}\left( breed_{others}\times calving_{medium} \right)+\beta_{14}\left( breed_{SIM}\times calving_{difficult} \right)+\beta_{15}\left( breed_{BS}\times calving_{difficult} \right) + \\ & \beta_{16}\left( breed_{others}\times calving_{difficult} \right)+\beta_{17}\left( parity_{2}\times season_{autumn} \right)+\beta_{18}\left( parity_{3+}\times season_{autumn} \right)+\beta_{19}\left( parity_{2}\times season_{winter} \right) + \\ & \beta_{20}\left( parity_{3+}\times season_{winter} \right)+\beta_{21}\left( parity_{2}\times season_{spring} \right)+\beta_{22}\left( parity_{3+}\times season_{spring} \right)+\beta_{23}\left( breed_{SIM}\times season_{autumn} \right) + \\ & \beta_{24}\left( breed_{BS}\times season_{autumn} \right)+\beta_{25}\left( breed_{others}\times season_{autumn} \right)+\beta_{26}\left( breed_{SIM}\times season_{winter} \right)+\beta_{27}\left( breed_{BS}\times season_{winter} \right) + \\ & \beta_{28}\left( breed_{others}\times season_{winter} \right)+\beta_{29}\left( breed_{SIM}\times season_{spring} \right)+\beta_{30}\left( breed_{BS}\times season_{spring} \right)+\beta_{31}\left( breed_{others}\times season_{spring} \right) \\ \alpha_{j} & \sim N\left( \gamma_{0}^{\alpha}+\gamma_{1}^{\alpha}\left( farm\_size_{medium} \right)+\gamma_{2}^{\alpha}\left( farm\_size_{large} \right)+\gamma_{3}^{\alpha}\left( calving_{medium}\times farm\_size_{medium} \right)+\gamma_{4}^{\alpha}\left( calving_{difficult}\times farm\_size_{medium} \right)+\gamma_{5}^{\alpha}\left( calving_{medium}\times farm\_size_{large} \right)+\gamma_{6}^{\alpha}\left( calving_{difficult}\times farm\_size_{large} \right),\sigma_{\alpha_{j}}^{2} \right)\text{, for farm\_id j = 1,}\ldots\text{,J} \end{matrix}$$

# 3 R code for scenarios

## 3.1 Worst case scenario

emmeans(m_multi_no_int, ~ breed,
 type ="response",
 weights = "prop",
 at = list(parity = "1", farm_size = "large", calving = "difficult", season = "winter"))

## breed prob SE df asymp.LCL asymp.UCL
## GH 0.483 0.0156 Inf 0.452 0.513
## SIM 0.391 0.0273 Inf 0.339 0.446
## BS 0.523 0.0459 Inf 0.433 0.611
## others 0.470 0.0211 Inf 0.429 0.512
##
## Confidence level used: 0.95
## Intervals are back-transformed from the logit scale

## 3.2 Worst case scenario

emmeans(m_multi_no_int, ~ breed,
 type ="response",
 weights = "prop",
 at = list(parity = "2", farm_size = "small", calving = "easy", season = "summer"))

## breed prob SE df asymp.LCL asymp.UCL
## GH 0.0189 0.00193 Inf 0.0154 0.0231
## SIM 0.0131 0.00127 Inf 0.0108 0.0158
## BS 0.0221 0.00380 Inf 0.0157 0.0309
## others 0.0180 0.00203 Inf 0.0144 0.0224
##
## Confidence level used: 0.95
## Intervals are back-transformed from the logit scale

## 3.3 All scenarios (not executed because 432 scenarios)

ref_grid(m_multi_no_int) %>%
 emmeans(~ breed+calving+parity+season+farm_size, weights = "prop", type = "response") %>%
 as_tibble() %>%
 arrange(desc(prob)) %>%
 select(-SE, -df)

# 4 Random Forest classification for PM which has two categories R code.

## 4.1 1) Without interactions

d_rf <- d %>%
 select(PM, breed, calving, farm_size, parity, season) %>%
 mutate(PM = factor(PM))

library(randomForest)

set.seed(1)
fit <- randomForest(PM ~ breed + parity + calving + season + farm_size,
 data = d_rf, importance = T, scale = T, mtry = 5, ntrees=10000, sampsize=c(1000,1000))

## 4.2 2) With interactions

library(vivid)
set.seed(101)
rf_fit <- vivi(fit = fit,
 data = d_rf,
 response = "PM",
 gridSize = 10,
 importanceType = "%IncMSE",
 nmax = 100,
 reorder = TRUE,
 class = 1,
 predictFun = NULL)

set.seed(1701)
importance_interactions <- viviNetwork(mat = rf_fit)

# 5 Brute-force R code

## 5.1 1) First, create the general function for mixed-effects models

glmer.glmulti<-function(formula,data,random="",...) {
 newf <- formula
 newf[[3]] <- substitute(f+r,
 list(f=newf[[3]],
 r=reformulate(random)[[2]]))
 glmer(newf,
 data=data,
 family = binomial(),
 nAGQ = 0,
 control = glmerControl(
 optimizer = "bobyqa",
 optCtrl = list(maxfun = 100000),
 check.conv.singular = .makeCC(action = "ignore", tol = 1e-9)),
 ...)
}

## 5.2 2) Secondly, run of all 32 models of all possible predictor combinations

- method = “d” checks how many models will be created
- method = “h” runs the models
- level = 1 runs models without interactions

glmulti(
 PM ~ breed + calving + parity + farm_size + season,
 random = "+(1|farm_id)",
 crit = aicc,
 data = d,
 method = "d",
 fitfunc = glmer.glmulti,
 marginality=F,
 level = 1)

## Initialization...
## TASK: Diagnostic of candidate set.
## Sample size: 133942
## 5 factor(s).
## 0 covariate(s).
## 0 f exclusion(s).
## 0 c exclusion(s).
## 0 f:f exclusion(s).
## 0 c:c exclusion(s).
## 0 f:c exclusion(s).
## Size constraints: min = 0 max = -1
## Complexity constraints: min = 0 max = -1
## Your candidate set contains 32 models.

## [1] 32

## 5.3 3) Finally, run all 1450 models of all possible combinations of 2nd order interactions.

- method = “d” checks how many models will be created
- method = “h” runs the models
- level = 2 runs models with interactions
- NOTE: no 3rd order interactions, e.g. parity*breed*season will be explored

glmulti(
 PM ~ breed + calving + parity + farm_size + season,
 random = "+(1|farm_id)",
 crit = aicc,
 data = d,
 method = "d",
 fitfunc = glmer.glmulti,
 marginality=F,
 level = 2)

## Initialization...
## TASK: Diagnostic of candidate set.
## Sample size: 133942
## 5 factor(s).
## 0 covariate(s).
## 0 f exclusion(s).
## 0 c exclusion(s).
## 0 f:f exclusion(s).
## 0 c:c exclusion(s).
## 0 f:c exclusion(s).
## Size constraints: min = 0 max = -1
## Complexity constraints: min = 0 max = -1
## Your candidate set contains 1450 models.

## [1] 1450

# 6 R packages used

report(sessionInfo()) %>%
 as.data.frame() %>%
 flextable() %>%
 bold(part = "header") %>%
 set_caption(caption = "R packages used in the study.") %>%
 width(j = "Reference", width = 4.9) %>%
 width(j = "Package", width = 1.1)

Table 6.1: R packages used in the study.

| **Package** | **Version** | **Reference** |
| --- | --- | --- |
| bookdown | 0.37 | Xie Y (2023). _bookdown: Authoring Books and Technical Documents with R Markdown_. R package version 0.37, <https://github.com/rstudio/bookdown>. Xie Y (2016). _bookdown: Authoring Books and Technical Documents with R Markdown_. Chapman and Hall/CRC, Boca Raton, Florida. ISBN 978-1138700109, <https://bookdown.org/yihui/bookdown>. |
| broom | 1.0.5 | Robinson D, Hayes A, Couch S (2023). _broom: Convert Statistical Objects into Tidy Tibbles_. R package version 1.0.5, <https://CRAN.R-project.org/package=broom>. |
| carData | 3.0.5 | Fox J, Weisberg S, Price B (2022). _carData: Companion to Applied Regression Data Sets_. R package version 3.0-5, <https://CRAN.R-project.org/package=carData>. |
| DataExplorer | 0.8.3 | Cui B (2024). _DataExplorer: Automate Data Exploration and Treatment_. R package version 0.8.3, <https://CRAN.R-project.org/package=DataExplorer>. |
| dlookr | 0.6.3 | Ryu C (2024). _dlookr: Tools for Data Diagnosis, Exploration, Transformation_. R package version 0.6.3, <https://CRAN.R-project.org/package=dlookr>. |
| dplyr | 1.1.4 | Wickham H, François R, Henry L, Müller K, Vaughan D (2023). _dplyr: A Grammar of Data Manipulation_. R package version 1.1.4, <https://CRAN.R-project.org/package=dplyr>. |
| effects | 4.2.2 | Fox J, Weisberg S (2019). _An R Companion to Applied Regression_, 3rd edition. Sage, Thousand Oaks CA. <https://socialsciences.mcmaster.ca/jfox/Books/Companion/index.html>. Fox J, Weisberg S (2018). "Visualizing Fit and Lack of Fit in Complex Regression Models with Predictor Effect Plots and Partial Residuals." _Journal of Statistical Software_, *87*(9), 1-27. doi:10.18637/jss.v087.i09 <https://doi.org/10.18637/jss.v087.i09>. Fox J (2003). "Effect Displays in R for Generalised Linear Models." _Journal of Statistical Software_, *8*(15), 1-27. doi:10.18637/jss.v008.i15 <https://doi.org/10.18637/jss.v008.i15>. Fox J, Hong J (2009). "Effect Displays in R for Multinomial and Proportional-Odds Logit Models: Extensions to the effects Package." _Journal of Statistical Software_, *32*(1), 1-24. doi:10.18637/jss.v032.i01 <https://doi.org/10.18637/jss.v032.i01>. |
| emmeans | 1.10.0 | Lenth R (2024). _emmeans: Estimated Marginal Means, aka Least-Squares Means_. R package version 1.10.0, <https://CRAN.R-project.org/package=emmeans>. |
| equatiomatic | 0.3.1 | Anderson D, Heiss A, Sumners J (2024). _equatiomatic: Transform Models into 'LaTeX' Equations_. R package version 0.3.1, https://datalorax.github.io/equatiomatic/, <https://github.com/datalorax/equatiomatic>. |
| flextable | 0.9.4 | Gohel D, Skintzos P (2023). _flextable: Functions for Tabular Reporting_. R package version 0.9.4, <https://CRAN.R-project.org/package=flextable>. |
| forcats | 1.0.0 | Wickham H (2023). _forcats: Tools for Working with Categorical Variables (Factors)_. R package version 1.0.0, <https://CRAN.R-project.org/package=forcats>. |
| ggforce | 0.4.2 | Pedersen T (2024). _ggforce: Accelerating 'ggplot2'_. R package version 0.4.2, <https://CRAN.R-project.org/package=ggforce>. |
| ggplot2 | 3.5.0 | Wickham H (2016). _ggplot2: Elegant Graphics for Data Analysis_. Springer-Verlag New York. ISBN 978-3-319-24277-4, <https://ggplot2.tidyverse.org>. |
| ggpubr | 0.6.0 | Kassambara A (2023). _ggpubr: 'ggplot2' Based Publication Ready Plots_. R package version 0.6.0, <https://CRAN.R-project.org/package=ggpubr>. |
| ggridges | 0.5.6 | Wilke C (2024). _ggridges: Ridgeline Plots in 'ggplot2'_. R package version 0.5.6, <https://CRAN.R-project.org/package=ggridges>. |
| glmulti | 1.0.8 | Calcagno V (2020). _glmulti: Model Selection and Multimodel Inference Made Easy_. R package version 1.0.8, <https://CRAN.R-project.org/package=glmulti>. |
| gridExtra | 2.3 | Auguie B (2017). _gridExtra: Miscellaneous Functions for "Grid" Graphics_. R package version 2.3, <https://CRAN.R-project.org/package=gridExtra>. |
| gtsummary | 1.7.2 | Sjoberg D, Whiting K, Curry M, Lavery J, Larmarange J (2021). "Reproducible Summary Tables with the gtsummary Package." _The R Journal_, *13*, 570-580. doi:10.32614/RJ-2021-053 <https://doi.org/10.32614/RJ-2021-053>, <https://doi.org/10.32614/RJ-2021-053>. |
| janitor | 2.2.0 | Firke S (2023). _janitor: Simple Tools for Examining and Cleaning Dirty Data_. R package version 2.2.0, <https://CRAN.R-project.org/package=janitor>. |
| kableExtra | 1.4.0 | Zhu H (2024). _kableExtra: Construct Complex Table with 'kable' and Pipe Syntax_. R package version 1.4.0, <https://CRAN.R-project.org/package=kableExtra>. |
| knitr | 1.45 | Xie Y (2023). _knitr: A General-Purpose Package for Dynamic Report Generation in R_. R package version 1.45, <https://yihui.org/knitr/>. Xie Y (2015). _Dynamic Documents with R and knitr_, 2nd edition. Chapman and Hall/CRC, Boca Raton, Florida. ISBN 978-1498716963, <https://yihui.org/knitr/>. Xie Y (2014). "knitr: A Comprehensive Tool for Reproducible Research in R." In Stodden V, Leisch F, Peng RD (eds.), _Implementing Reproducible Computational Research_. Chapman and Hall/CRC. ISBN 978-1466561595. |
| leaps | 3.1 | Miller TLboFcbA (2020). _leaps: Regression Subset Selection_. R package version 3.1, <https://CRAN.R-project.org/package=leaps>. |
| lme4 | 1.1.35.1 | Bates D, Mächler M, Bolker B, Walker S (2015). "Fitting Linear Mixed-Effects Models Using lme4." _Journal of Statistical Software_, *67*(1), 1-48. doi:10.18637/jss.v067.i01 <https://doi.org/10.18637/jss.v067.i01>. |
| lmerTest | 3.1.3 | Kuznetsova A, Brockhoff PB, Christensen RHB (2017). "lmerTest Package: Tests in Linear Mixed Effects Models." _Journal of Statistical Software_, *82*(13), 1-26. doi:10.18637/jss.v082.i13 <https://doi.org/10.18637/jss.v082.i13>. |
| lubridate | 1.9.3 | Grolemund G, Wickham H (2011). "Dates and Times Made Easy with lubridate." _Journal of Statistical Software_, *40*(3), 1-25. <https://www.jstatsoft.org/v40/i03/>. |
| Matrix | 1.6.5 | Bates D, Maechler M, Jagan M (2024). _Matrix: Sparse and Dense Matrix Classes and Methods_. R package version 1.6-5, <https://CRAN.R-project.org/package=Matrix>. |
| modelbased | 0.8.7 | Makowski D, Ben-Shachar M, Patil I, Lüdecke D (2020). "Estimation of Model-Based Predictions, Contrasts and Means." _CRAN_. <https://github.com/easystats/modelbased>. |
| officer | 0.6.5 | Gohel D, Moog S (2024). _officer: Manipulation of Microsoft Word and PowerPoint Documents_. R package version 0.6.5, <https://CRAN.R-project.org/package=officer>. |
| performance | 0.10.9 | Lüdecke D, Ben-Shachar M, Patil I, Waggoner P, Makowski D (2021). "performance: An R Package for Assessment, Comparison and Testing of Statistical Models." _Journal of Open Source Software_, *6*(60), 3139. doi:10.21105/joss.03139 <https://doi.org/10.21105/joss.03139>. |
| purrr | 1.0.2 | Wickham H, Henry L (2023). _purrr: Functional Programming Tools_. R package version 1.0.2, <https://CRAN.R-project.org/package=purrr>. |
| R | 4.3.2 | R Core Team (2023). _R: A Language and Environment for Statistical Computing_. R Foundation for Statistical Computing, Vienna, Austria. <https://www.R-project.org/>. |
| randomForest | 4.7.1.1 | Liaw A, Wiener M (2002). "Classification and Regression by randomForest." _R News_, *2*(3), 18-22. <https://CRAN.R-project.org/doc/Rnews/>. |
| readr | 2.1.5 | Wickham H, Hester J, Bryan J (2024). _readr: Read Rectangular Text Data_. R package version 2.1.5, <https://CRAN.R-project.org/package=readr>. |
| readxl | 1.4.3 | Wickham H, Bryan J (2023). _readxl: Read Excel Files_. R package version 1.4.3, <https://CRAN.R-project.org/package=readxl>. |
| report | 0.5.8 | Makowski D, Lüdecke D, Patil I, Thériault R, Ben-Shachar M, Wiernik B (2023). "Automated Results Reporting as a Practical Tool to Improve Reproducibility and Methodological Best Practices Adoption." _CRAN_. <https://easystats.github.io/report/>. |
| rJava | 1.0.11 | Urbanek S (2024). _rJava: Low-Level R to Java Interface_. R package version 1.0-11, <https://CRAN.R-project.org/package=rJava>. |
| sjPlot | 2.8.15 | Lüdecke D (2023). _sjPlot: Data Visualization for Statistics in Social Science_. R package version 2.8.15, <https://CRAN.R-project.org/package=sjPlot>. |
| stringr | 1.5.1 | Wickham H (2023). _stringr: Simple, Consistent Wrappers for Common String Operations_. R package version 1.5.1, <https://CRAN.R-project.org/package=stringr>. |
| tibble | 3.2.1 | Müller K, Wickham H (2023). _tibble: Simple Data Frames_. R package version 3.2.1, <https://CRAN.R-project.org/package=tibble>. |
| tidyr | 1.3.1 | Wickham H, Vaughan D, Girlich M (2024). _tidyr: Tidy Messy Data_. R package version 1.3.1, <https://CRAN.R-project.org/package=tidyr>. |
| tidyverse | 2.0.0 | Wickham H, Averick M, Bryan J, Chang W, McGowan LD, François R, Grolemund G, Hayes A, Henry L, Hester J, Kuhn M, Pedersen TL, Miller E, Bache SM, Müller K, Ooms J, Robinson D, Seidel DP, Spinu V, Takahashi K, Vaughan D, Wilke C, Woo K, Yutani H (2019). "Welcome to the tidyverse." _Journal of Open Source Software_, *4*(43), 1686. doi:10.21105/joss.01686 <https://doi.org/10.21105/joss.01686>. |
| vivid | 0.2.8 | Inglis A, Parnell A, Hurley CB (2022). "Visualizing Variable Importance and Variable Interaction Effects in Machine Learning Models." _Journal of Computational and Graphical Statistics_, 1-13. |
