# Supplementary material for: Perinatal mortality in German dairy cattle: Unveiling the importance of cow-level risk factors and their interactions using a multifaceted modelling approach
Source: PLoS One. 2024 Apr 17;19(4):e0302004. doi: 10.1371/journal.pone.0302004 (PMC11023303; doi:10.1371/journal.pone.0302004)
Supplement: Attachment — Submitted filename: comments reviewer.docx [file pone.0302004.s002.docx]

General comment:
The authors address an important topic and use adequate vocabulary to report their study. Perinatal mortality is a welfare issue and currently below target. I cannot assess all information due to very low resolution in the images (at least in the version I obtained). Also I have a hard time understanding what happened during data collection and model building. I cannot assess the raw data quality. Also, I find it difficult to navigate through potential contradictions (for instance when it comes to the level of the analysis). There is little information on model architecture, at least the statistical equations should be printed in a paper whose aims include comparison of methods. It is unfortunate, that much space in the manuscript is used for repetition (see comments to discussion sections), whereas information for someone who reads your paper for the first time lacks. Please revise.

L 18, 44: There are no necessary calf losses, please delete ‘unnecessary’.

L 20: Can knowledge be comparable? Please revise style.

L 33: I reckon you investigated associations, not causalities. Please consider revising “influential factors”.

L38: Your abstract lacks two or three discussion-like sentences concerning a) veterinary content and b) statistical methodological findings. This is also a problem in the discussion section itself, where interpretation is lacking.

L92 Please add that Saxony and Thuringia are political units/regions/provinces if this is the case and relevant.

L 115: Classic log reg may be misleading in a multi-farm setting, not mixed log reg. Please carefully distinguish the model names as here it is unclear which model use mean (classic vs. log reg).

L 131: Please provide a detailed sample size calculation here, including software used and name of test.

L136: We haven’t learnt about regional division of the study team yet. Why didn’t you use the national farm registration database in Bavaria?

L138: How was this response rate distributed across regions? It is not convenient to go to other articles to understand crucial information of the present one.

L143: How many researchers were there in total and per region? I acknowledge that you need various staff members to cover the farms across regions. A more detailed insight into potential observation biases will be helpful.

L153: Please briefly describe this procedure here.

L155: This is a result and needs to go to the results sections. How many farmer responded and how many were excluded?

L155ff: dito

L 174: Provide information on gestation length data quality in order to be able to follow your approach.

L197: Please add seasons’ cutoffs in months or weeks.

L207: What did qualify a variable to be offered to the model? Did you perform univariate analysis first or did you add all variables per se?

L207: Did your models fail to converge when not using BOBYQA optimizer?

L207: Did you check model assumptions for logistic models besides multicollinearity? How did you handle outliers? Did you check linearity of the logit for those variables that were continuous?

L209: Which factors did you consider to be possible confounding factors? Did you offer all interaction combinations as default? It is not clearly state in my opinion and thus hard to follow.

L225: Did you mention that you conducted your analysis at the cow-level any earlier than that? I believe you did not. Please specify this in introduction and abstract. I also suggest to specify this in the title. You use also non-cow-level such as season. This leaves me somewhat puzzled as to which is the level of the model. I understand that you account for the hierarchical structure of the data at the farm level. Please clarify.

L256: …with different methods than what?

L259: Earlier you state that models without interaction terms are more prone to confounding. Why did you now do model without interaction terms? This is not clear to me. Compare with L 3034.

L262: The variable breed is strongly unequally distributed among categories. Please discuss to what extent this may have biased your results and how you accounted for it. Does this effect comparability within your study, i.e., do you think it is appropriate to compare data from ~115,000 GH to ~1,300 BS?

L 263: Please provide mean and sd for PM across farms.

L 268: dito concerning distribution of the data among categories. How did you handle (relative) low numbers in calving ease? How did this influence model building?

L 285-288: Please adhere to scientific English rather than colloquial English through use of parallel structure, i.e., name variables which are significantly associated first for better readability.

Fig 4.1.: The low resolution of the figures is inconvenient, especially in Fig 4.2. I do not know if this happened automatically/unintentionally when submitting or is a bug from the submission tool. I cannot read it and therefore cannot assess it.

L303-307: This is unclear to me. Please explain. Also, do you mean ‘predictors’ or ‘potential predictors’?

L323-324: How did you calculate this? Please provide guidance, a code snippet or explanations on this.

Table 4.2.: What is the advantage of combining predictors (at the cow-level)? It reads as if a cow you be attributed to two breeds or as if you grouped calvings of an individual pluriparous cow. Would you please elaborate on this? Are the ‘/’ indicating reference levels? There is information missing to understand your results when going through the article with a perspective from a first reader.

L 453f: Are these absolute or relative percentage values? Where did you report this data in the manuscript?

L527: I understand your argumentation, however, have a hard time believing that any risk factor applies to all individuals of all populations. To me, your sentence reads like this. I would appreciate you weakened that statement to something alike “not an important/predominant predictor”.

General remark to the discussion: I miss a discussion on data quality of the raw data. Do you think there is little entry errors when it comes to parity or calving ease? What about inter- and intraobserver reliability when there is hundreds of farmers involved? I appreciate your huge dataset but would be more cautious when interpreting this data. How is data entered into the system? What is the maximal delays for data entry?

L581f: This should be based on suitable literature as it reads somewhat as a speculation.

L591: You shouldn’t be repeating your results and methods during discussion. This is actually making your paper lengthy and blurs new information among already given information. This is a general style remark also applying to earlier parts of the discussion.

L608: dito

L643: dito. Please revise the discussion section and put emphasis on what your results *mean to the reader, to the cows, to the future (of PM) etc* and refrain from giving the same information from M&M or results in other words. Should we prioritize SIM breeds over GH in those German regions now, due to increased PM? Is the trade-off between a likely higher average milk yield and more dead calves acceptable? If yes, why? Should we transition to seasonal calvings to summer? If not, why? What type of programs could be deployed to minimize PM? As experts in the field, do you think that there is practical approaches beyond theory that may work?

L 642/L205/255ff: The level of analysis is farm and not animal or both? Why didn’t you include a random effect for animal? Didn’t your dataset offer several calvings from the same individual?

L681: For me as an intermediate in veterinary statistics, it is not comprehensible from your explanations how you transitioned from unbalanced to the balances RF model. I think the great majority of your readers would appreciate you elaborated on this in more detail.

L685: Backwards selection does not work through adding of variables. Please proof me wrong or delete.
