# Supplementary material for: Perinatal mortality in German dairy cattle: Unveiling the importance of cow-level risk factors and their interactions using a multifaceted modelling approach
Source: PLoS One. 2024 Apr 17;19(4):e0302004. doi: 10.1371/journal.pone.0302004 (PMC11023303; doi:10.1371/journal.pone.0302004)
Supplement: Attachment — Submitted filename: comments_zablotski_perinatalmortality.docx [file pone.0302004.s003.docx]

This interesting paper is well written. Specific comments are found below mostly surrounding clarification.

Title

Please, make the title more compact and attractive. In my opinion, it is not necessary to add the methods used (this is information for the Materials & Methods section). The title should spark the reader's interest.

Abstract

You've outlined the two aims of the study. While you successfully discuss the first aim, the description of results and conclusions for the second aim, specifically the evaluation and comparison of different statistical methods, is currently missing. Could you please elaborate on this aspect with one or two additional sentences?

Introduction

At line 99, you give the number of calving records, but in my opinion, this is for the Material and Methods section.

Lines 107-110 should be relocated to precede the aims of the study, as they provide essential context. Additionally, lines 110-120 should be moved to the materials and methods section for better organizational coherence.

Materials & methods

Line 180-181: Place figure captions in the manuscript text in read order, immediately following the paragraph where the figure is first cited. So, please, replace this caption to line 134.

Line 136: provide the time period of the study please.

Line 138: Could you clarify the intended meaning of a response rate 'between 6 and 9%'? It would be helpful to provide the overall global response rate first and then specify the response rates for each involved region to offer a more detailed breakdown.

Line 156: In the literature, perinatal mortality typically refers to stillbirths beyond day 260 of gestation and calves that died within 48 hours after birth. However, the text is not entirely clear on whether the included cases encompass only full-term stillbirths and deaths within 48 hours postpartum, or if it also includes stillbirths that occurred between 260 days and full term. Please provide clarification on this point.

Line 156-157: Why were observed abortions not included in this definition? The current inclusion seems to result in a potential overestimation of the perinatal mortality rate. Please provide clarification on the rationale behind this decision?

Line 173: In the earlier section of the manuscript, the term 'number of calves per birth' was used. For consistency, please retain the same variable name throughout and replace 'litter size' with 'number of calves per birth.

Line 174: please, provide information on how many multiple births were excluded.

Line 175-176: Was crown-rump length measured to estimate the age of the fetus/calf at stillbirth? If so, could you please describe the methodology used for this measurement and provide relevant details? If not, could you provide an explanation for the omission of this measurement and the chosen alternative, if any?

Line 182: why was the number of abortions not recorded? Please, explain this more in detail.

Line 186: Please provide the complete written form for abbreviations the first time they are used.

Line 193-195: I have reservations about the classification of calving ease. For instance, categorizing a caesarean section as difficult may not accurately reflect cases where the procedure's difficulty is potentially lower than instances involving multiple helpers and mechanical pulling tools. Could you clarify whether all caesarean sections are performed after attempting mechanical pulling or with assistance from several helpers? If not, should it be better to create a fourth category (calving after caesarean section)? It may be advisable to consider excluding cases involving fetotomy, as in most instances, mortality is not directly attributed to the fetotomy procedure itself. Typically, fetotomy is performed due to the death of a large calf that cannot be delivered manually. Would you agree?

Line 208: Change 'p-value' to 'P value' (italicized with capital) throughout the entire manuscript. Please review the entire document for this correction.

Line 216: what do you mean with “explanatory” and “R^2^”? I suppose this is “conditional coefficient of determination R2” and “Marginal coefficient of determination” respectively? Please, make this more clear.

Line 219: please, mention how many were others in region South.

Line 223: please change > into ≥

Line 224-225. The information is partially available in lines 217-218. Please add details about others in the South at line 219.

Line 228: Please make it easier to read by choosing either 'differences' or 'contrasts' and removing the other.

Line 251-253: please, provide a reference for this.

Results

Line 294: in figure 4.1d, it looks like calving ease and parity have the same predictive value. Please provide the values in the text, to make this clearer.

Line 335: if significantly, please provide *P* value.

Line 373: please provide *P* value

Line 374-375: please, rephrase this sentence, and provide the same information for each group.

Line 376-378: is this significant? If so, please provide *P* values.

Line 378: do you mean rate or incidence or prevalence? Please check this.

Line 381: please provide *P* value

Line 387-391: are these results statistically significant? If not, you can not say “higher” or “lower”, but you should say “is numerically lower, although not significantly”. If it is significant, please provide *P* values.

Line 393-404: provide P values were necessary (when results were statistically significant).

Discussion

In my opinion, it is not necessary to divide the discussion into separated subsections.

Line 440: I find it somewhat confusing that you state your study aligns with the findings of Voljc et al., but at line 442, you mention that the results for Brown Swiss deviate from this trend. Could you please provide clarification to make this aspect clearer?

In each subsection, begin by presenting your main findings. Concentrate on highlighting your results first, and subsequently, engage in a discussion comparing your findings with those of relevant studies.

I noticed the absence of a general conclusion for the study. Could you please provide a brief conclusion that encapsulates key findings and highlights elements pertinent for other researchers and field workers in your area, as well as in other regions globally?

Figure 4.1. and 4.2.: what do the asterisks mean? Explain in the caption please.

Figure 4.2.: what is Vint and Vimp? Please, explain this in the caption.
